# Effects of Supplementary Blue and UV-A LED Lights on Morphology and Phytochemicals of *Brassicaceae* Baby-Leaves

**DOI:** 10.3390/molecules25235678

**Published:** 2020-12-02

**Authors:** Yamin Li, Yinjian Zheng, Dongqiang Zheng, Yiting Zhang, Shiwei Song, Wei Su, Houcheng Liu

**Affiliations:** 1 College of Horticulture, South China Agricultural University, Guangzhou 510642, China; yaminli@stu.scau.edu.cn (Y.L.); zhengdongqiang@guangd.picc.com.cn (D.Z.); yitingzhang@scau.edu.cn (Y.Z.); swsong@scau.edu.cn (S.S.); susan_l@scau.edu.cn (W.S.); 2 Institute of Urban Agriculture, Chinese Academy of Agricultural Sciences, Chengdu 610299, China; zhengyinjian@caas.cn

**Keywords:** *Brassicaceae* baby-leaves, blue light, UV-A light, biomass, morphology, glucosinolates, antioxidants, minerals

## Abstract

*Brassicaceae* baby-leaves are good source of functional phytochemicals. To investigate how Chinese kale and pak-choi baby-leaves in response to different wavebands of blue (430 nm and 465 nm) and UV-A (380 nm and 400 nm) LED, the plant growth, glucosinolates, antioxidants, and minerals were determined. Both agronomy traits and phytochemical contents were significantly affected. Blue and UV-A light played a predominant role in increasing the plant biomass and morphology, as well as the contents of antioxidant compounds (vitamin C, vitamin E, phenolics, and individual flavonols), the antioxidant activity (DPPH and FRAP), and the total glucosinolates accumulation. In particular, four light wavebands significantly decreased the content of progoitrin, while 400 nm UV-A light and 430 nm blue light were efficient in elevating the contents of sinigrin and glucobrassicin in Chinese kale. Meanwhile, 400 nm UV-A light was able to increase the contents of glucoraphanin, sinigrin, and glucobrassicin in pak-choi. From the global view of heatmap, blue lights were more efficient in increasing the yield and phytochemical levels of two baby-leaves.

## 1. Introduction

Chinese kale (*Brassica alboglabra* Bailey) and pak-choi (*Brassica campestris* L. ssp. *chinensis* var. *communis*) are native to China, distributing widely in South China and Southeast Asia. These *Brassicaceous* vegetables are famous due to their rich contents of phytonutrients, including sulphoraphane, anthocyanins, glucosinolates (GLs), carotenoids, flavonoids and general antioxidants. Baby-leaves are harvested from vegetable or herb seedlings, with three to five true leaves. Considerable evidence indicated the health and nutritional benefits associated with the consumption of fresh-cut baby-leaves [1]. The younger plants have excellent flavor and higher nutritional abundance than the mature ones, the products of Chinese kale and pak-choi baby-leaves have become popular in the market.

Natural antioxidants and bio-active compounds have health-promoting or disease-preventing properties [2,3,4,5,6]. Vitamin C has been proved to scavenge superoxide and help the regeneration of vitamin E and glutathione [4,7]. Carotenoids are mainly responsible for the yellow to red colors in plants [8], which function as the light-harvesting pigments in chloroplasts [9,10] and commercially related to the food and cosmetic industries. Except for fruits and flowers, carotenoids are also synthesized in the photosynthetic organs like the sink tissue, as well as the reserve roots of plants [11]. For health concerning, carotenoids act as the quenchers of free radicals and singlet oxygen and prevent human from vitamin A deficiency [12]. Phenolics and flavonoids have anti-inflammatory and cardiovascular diseases prevention properties [13]. Anthocyanins are pigments that accumulate in plant vacuoles and have powerful antioxidant activities [14,15]. The hydrolytic products of GLs have multiple functions, such as cancer chemoprevention [16,17,18,19,20,21,22], antibacterial, anti-corrosion [23], and moderating neuropathic pain [24]. The intake of minerals also helps maintaining human body health. Besides, it is considered safer and more acceptable to ingest nutrients and antioxidants derived from vegetables in the daily diet.

Various attempts have been conducted to regulate the growth and nutritional quality of vegetables, most commonly by artificially altered environment conditions [25]. Light environment is one of the pivotal determinants that drives photosynthesis, growth, development, and metabolism. Control of the light regime offers possibilities for improving the production and quality of sprouts [26]. For example, additional blue light alleviated the suppressions in photosystem activity and electron transport capacity caused by monochromatic red light in cucumber leaves [27], and accelerated biosynthesis of flavonoids and phenolics in basil seedlings [28]. Merely 16 h blue light supplementation significantly increased the antioxidant capacity of lettuce seedlings [29]. The application of UV-A light enhanced the accumulation of several phytochemicals in broccoli sprouts, including gallic acid, kaempferol glucoside, lutein, chlorophylls, and neoxanthin, while UV-B light increased the contents of aliphatic and indole GLs [30]. Meanwhile, UV-A light could improve the brightness and chroma of broccoli [31] and maintain the biomass of Brussels sprouts [32]. Moreover, vegetables accumulate minerals according to their requirements, and different light spectra have significant effects on the accumulation of different minerals in biological systems [33].

So far, many studies have focused on increasing the yield and quality of baby-leaf vegetables by changing light intensity, photoperiod, and light quality combinations. However, there were fewer reports on the effects of specific light wavebands on the growth and phytochemicals of baby-leaf vegetable cultivation. In this study, how blue (430 nm and 460 nm) and UV-A (380 nm and 400 nm) LED light affected the agronomy traits and functional phytochemical levels in Chinese kale and pak-choi baby-leaves was investigated. 

## 2. Results

### 2.1. Agronomy Traits under Supplementary Different Wavebands of Blue and UV-A Light

**Figure 1:**
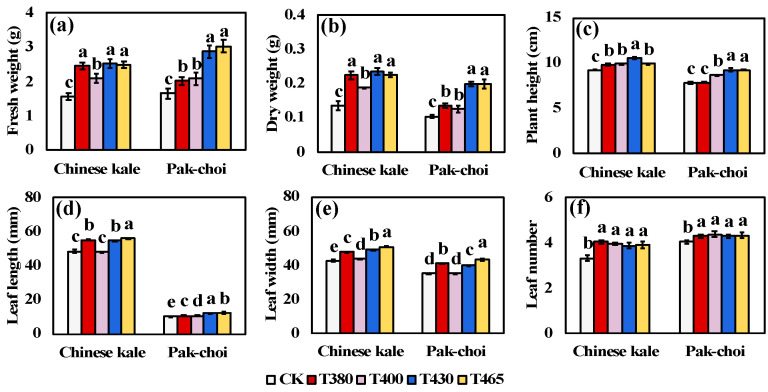
Agronomy traits of Chinese kale and pak-choi baby-leaves under supplementary different wavebands of blue and UV-A light. The fresh weight (**a**), dry weight (**b**), plant height, (**c**) leaf length (**d**), leaf width (**e**), and leaf number (**f**) of two baby-leaves. Different letters (a–e) upon the bar plots indicate significant difference at *p* < 0.05 using one-way analysis of variance with Duncan’s multiple-range test.

The plant biomass and morphological indices of two baby-leaves were significant promoted by blue and UV-A light supplementation (Figure 1). The leaf number of two baby-leaves significant increased under all light wavebands (Figure 1f), indicating an accelerated growth of young plants. The highest plant height, the largest leaf length and leaf width were observed in T430 and/or T465, followed by T380 and T400 (Figure 1c–e), which in turn contributed to the increase in biomass. Compared to CK, the fresh weight of Chinese kale and pak-choi were highly promoted by T430 (62.22, 73.79%) and T465 (60.08, 83.47%), followed by T380 (58.21, 22.38%) and T400 (34.42, 26.01%) (Figure 1a), and the dry weight increased by T430 (74.08, 92.23%), T465 (66.66, 91.47%), T380 (65.43, 30.55%) and T400 (39.38, 20.56%) (Figure 1b). In terms of the agronomy traits, the promotive effects of blue light were better than UV-A light.

### 2.2. Phytochemicals under Supplementary Different Wavebands of Blue and UV-A Light

**Figure 2:**
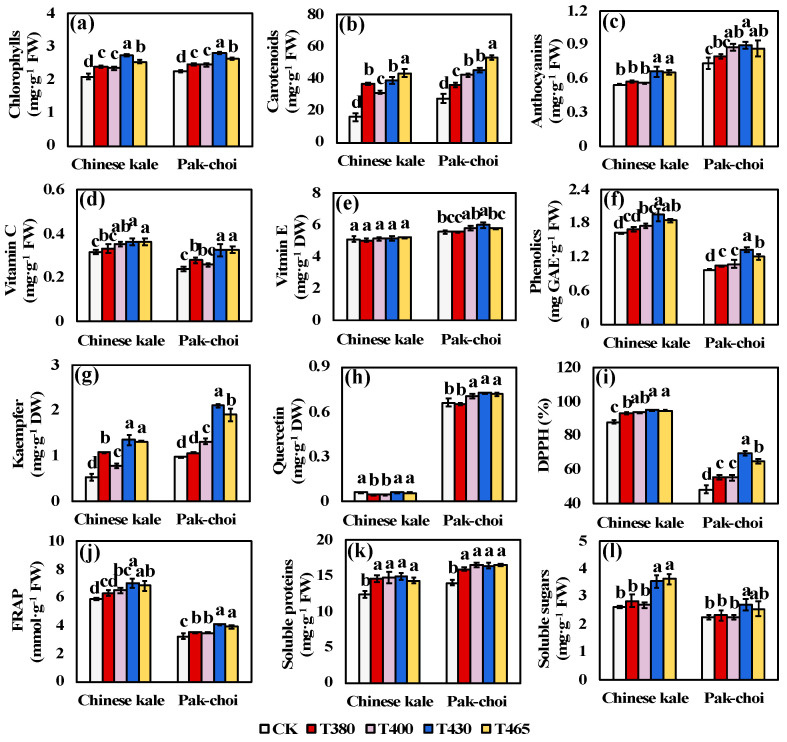
Phytochemical profiles of two baby-leaves under different wavebands of blue and UV-A light. The contents of (**a–c**) pigments, (**d**–**h**) antioxidant compounds and (**k**–**l**) nutritional compounds. (**i**–**j**) were the antioxidant activities. DPPH = 2,2-diphenyl-1-picrylhydrazyl radical scavenging, FRAP = ferric reducing antioxidant power. Different letters (a–e) upon the bar plots indicate significant difference at *p* < 0.05 using one-way analysis of variance with Duncan’s multiple-range test.

Chlorophylls, carotenoids and anthocyanins are the main types of pigments distributed in plants, and they also work as excellent antioxidants and benefit human health. The contents of chlorophylls, carotenoids, and anthocyanins were greatly increased by supplementary blue and UV-A wavebands, except that UV-A maintained the similar level of anthocyanins in Chinese kale (Figure 2a–c). T430 and/or T465 led to the highest pigment contents in both two baby-leaves, followed T380 and T400.

Similarly, in both two baby-leaves, the antioxidant compounds such as vitamin C, phenolics and kaempfer, and the antioxidant activities such as DPPH and FRAP showed a stronger enhancement by T430 and T465, and relatively weaker increase by T380 and T400 (Figure 2d,f,g,i,j). For vitamin E, no significant change was detected in Chinese kale under different wavebands (Figure 2e), while, in pak-choi, the vitamin E content was significantly increased as other antioxidants (Figure 2e). Moreover, the content of quercetin decreased by T380 and T400 in Chinese kale, while increased by T400, T430 and T465 in pak-choi (Figure 2h).

Soluble proteins and soluble sugars played important role in vegetable stress resistance, as well as flavor substance and nutrition quality. In both two baby-leaves, all supplementary light wavebands significantly increased the content of soluble proteins, while UV-A did not affect the content of soluble sugars (Figure 2k,l). Additionally, blue light markedly enhanced the soluble sugar accumulation in Chinese kale, but only T430 increased the content in pak-choi (Figure 2l).

Overall, the blue wavebands were superior in elevating the phytochemicals in Chinese kale and pak-choi than UV-A wavebands. In addition, the differential results observed between two baby-leaves implied that the regulatory effects also dependent on plant species.

### 2.3. Glucosinolates under Supplementary Different Wavebands of Blue and UV-A Light

**Figure 3:**
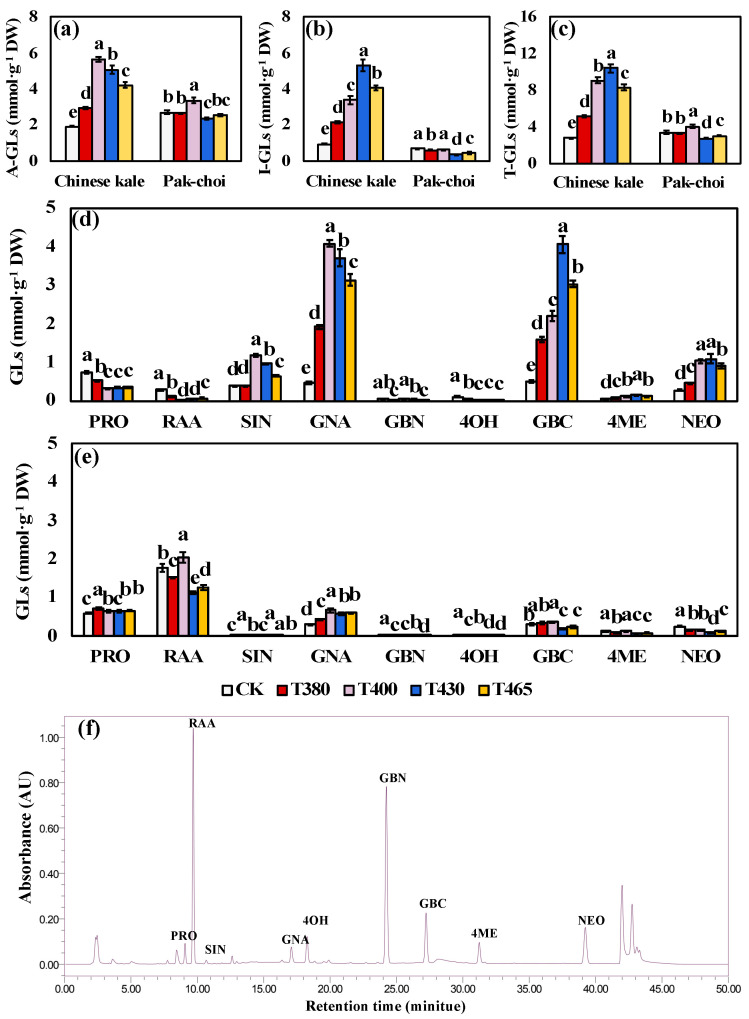
Glucosinolate of two baby-leaves under different wavebands of blue and UV-A light. The contents of (**a**) aliphatic, (**b**) indolic, and (**c**) total glucosinolates. The individual glucosinolate content in (**d**) Chinese kale and (**e**) pak-choi. Identification of nine glucosinolates (**f**) from high performance liquid chromatography. A-GLs = aliphatic flucosinolates, I-GLs = indolic glucosinolates, T-GLs = total glucosinolates, PRO = progoitrin, RAA = glucoraphanin, SIN = sinigrin, GNA = gluconapin, GBN= glucobrassicanapin, 4OH = 4-hydroxyglucobrassicin, GBC = glucobrassicin, 4ME = 4-methoxyglucobrassicin, NEO = neoglucobrassicin. Different letters (a–e) upon the bar plots indicate significant difference at *p* < 0.05 using one-way analysis of variance with Duncan’s multiple-range test.

Nine individual glucosinolates were identified and quantified in both Chinese kale and pak-choi baby-leaves, including five aliphatic GLs (A-GLs) [progoitrin (PRO), glucoraphanin (RAA), sinigrin (SIN), gluconapin (GNA), glucobrassicanapin (GBN)] and four indolic GLs (I-GLs) [4-hydroxyglucobrassicin (4OH), glucobrassicin (GBC), 4-methoxyglucobrassicin (4ME), neoglucobrassicin (NEO)] (Figure 3f). Regarding baby-leaf cultivars (based on CK), the content of I-GLs was higher in Chinese kale, whereas the contents of A-GLs and total GLs (T-GLs) were higher in pak-choi (Figure 3a–c). Interestingly, in Chinese kale, the contents of A-GLs, I-GLs, and T-GLs were drastically enhanced by blue and UV-A supplementation: the A-GLs significantly increased under T400 (194.26%), T430 (163.96%), T465 (119.04%), and T380 (53.88%); the I-GLs drastically enhanced by T430 (456.37%), T465 (326.24%), T400 (254.93%), and T380 (129.47%); the T-GLs increased under T430 (260.91%), T400 (214.38%), T465 (187.75%), and T380 (78.94%). Whereas, in pak-choi, these contents were mostly down-regulated: except for T400 which increased the A-GLs (24.25%) and T-GLs (18.72%), other light wavebands significantly or somewhat retarded the accumulation of A-GLs (T430, −11.94%), I-GLs (T380, −10.50%; T465, −32.93%; T430, −45.67%), and T-GLs (T380, −2.87%; T465, 11.54%; T430, −18.87%). These results indicated a differential sensitivity of GLs between two baby-leaves (Figure 3a–c).

Besides, the predominant individual GLs in two baby-leaves presented difference. Without light supplementation, the most abundant three GLs in Chinese kale were PRO (0.75 mmol·g^−1^ DW), GBC (0.51 mmol·g^−1^ DW), and GNA (0.46 mmol·g^−1^ DW) (Figure 3d), and those in pak-choi were RAA (1.78 mmol·g^−1^ DW), PRO (0.60 mmol·g^−1^ DW), and GBC (0.32 mmol·g^−1^ DW) (Figure 3e). In Chinese kale supplemented with different light wavebands, the GNA and GBC contents rapidly reached the highest level, followed by SIN and NEO (Figure 3d), while, the contents of PRO, RAA and 4OH were down-regulated by different wavebands (Figure 3d). In pak-choi, the effects of supplementary different wavebands on GLs were different. The RAA and GBC contents increased under T400, while decreased or unchanged under other treatments (Figure 3e). The GNA content increased in all light treatments, while the NEO content declined (Figure 3e).

### 2.4. Minerals under Supplementary Different Wavebands of Blue and UV-A Light

**Figure 4:**
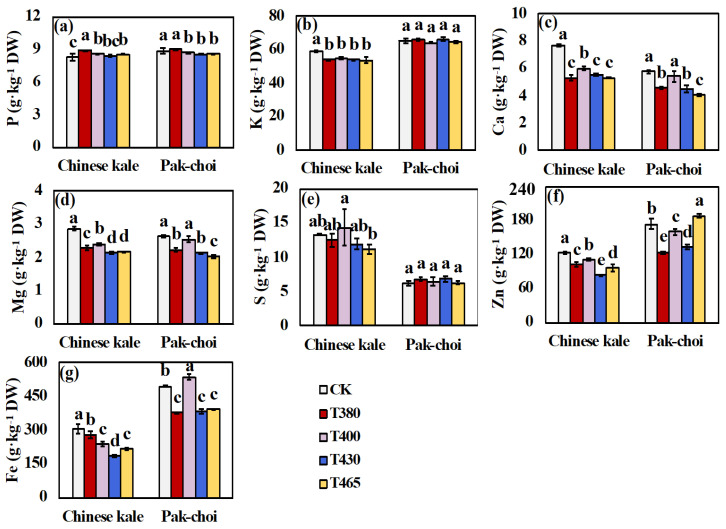
Minerals of two baby-leaves under different wavebands of blue and UV-A light. The contents of (**a**) P = phosphorous, (**b**) K = potassium, (**c**) Ca = calcium, (**d**) Mg = magnesium, (**e**) S = sulphur, (**f**) Zn = zinc, and (**g**) Fe = iron. Different letters (a–e) upon the bar plots indicate significant difference at *p* < 0.05 using one-way analysis of variance with Duncan’s multiple-range test.

The minerals [phosphorous (P), potassium (K), calcium (Ca), magnesium (Mg), sulphur (S), zinc (Zn), iron (Fe)] of Chinese kale and pak-choi baby leaves were significantly influenced by supplementary blue and UV-A lights (Figure 4). In Chinese kale, the contents of K, Ca, Mg, Zn and Fe markedly decreased in all treatments. Meanwhile, P content was higher under T380, T 400 and T465, and S was found no change, respectively compared to CK (Figure 4 a,e). In pak-choi, the contents of K and S were less affected by blue and UV-A light. The contents of P, Ca, Mg and Fe declined under UV-A light (T430 and T465), while Zn and Fe increased in T465 and T400, respectively.

### 2.5. Heatmap Analysis

**Figure 5:**
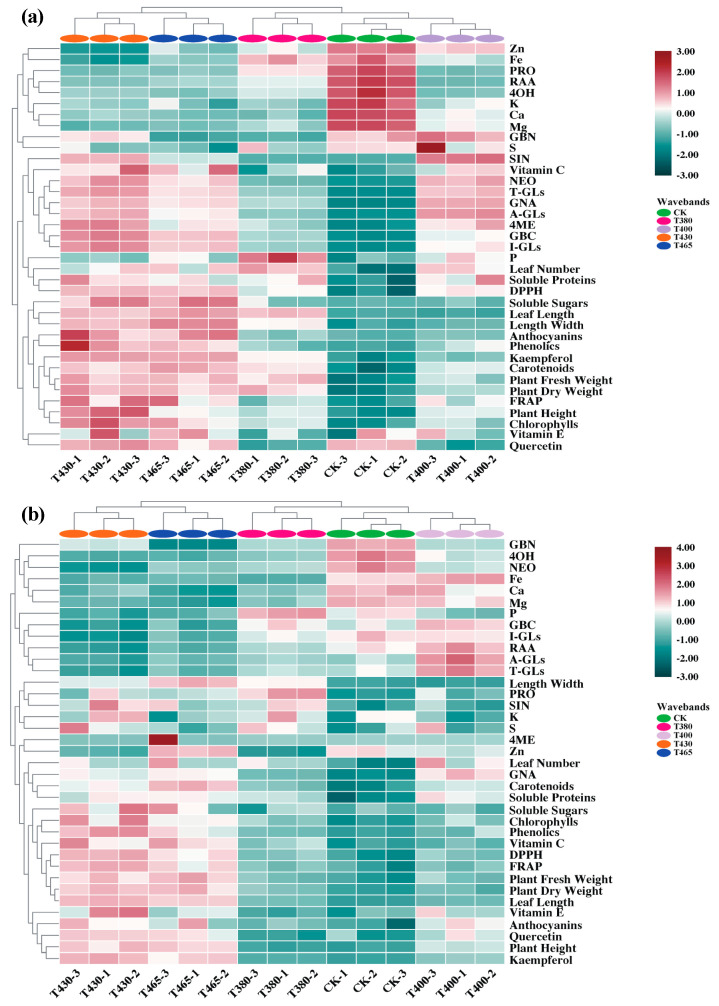
Cluster heatmap analysis summarizing two baby-leaves response to supplementary blue and UV-A wavebands. (**a**) Chinese kale and (**b**) Pak-choi response patterns. Results are visualized using a false color scale with red indicating an increased parameter, and green indicating a decreased parameter. DPPH = 2,2-diphenyl-1-picrylhydrazyl radical scavenging, FRAP = ferric reducing antioxidant power, A-GLs = aliphatic flucosinolates, I-GLs = indolic glucosinolates, T-GLs = total glucosinolates, PRO = progoitrin, RAA = glucoraphanin, SIN = sinigrin, GNA = gluconapin, GBN= glucobrassicanapin, 4OH = 4-hydroxyglucobrassicin, GBC = glucobrassicin, 4ME = 4-methoxyglucobrassicin, NEO = neoglucobrassicin, P = phosphorous, K = potassium, Ca = calcium, Mg = magnesium, S = sulphur, Zn = zinc, Fe = iron.

A heatmap synthesized the response of agronomy traits, phytochemicals, GLs, and minerals, providing an integrated view of the effect of supplementary different blue and UV-A wavebands on Chinese kale and pak-choi baby-leaves (Figure 5).

Concerning Chinese kale, all light wavebands were divided into two main clusters. One was the pair of cluster CK and cluster T400, and they were equidistant from cluster T380, which was characterized by considerably higher contents of minerals such as Zn, Fe, K, Ca, Mg, and S, as well as three aliphatic GLs (PRO, RAA, and GBN) and one indolic GLs (4OH) (Figure 5a). The other was the pair of cluster T430 and cluster T465, which was distinguished by excellent growth performance (fresh weight, dry weight, leaf length, leave width, and leaf number), richer contents of antioxidant compounds (Vitamin C, anthocyanins, phenolics, kaempferol, carotenoids, Vitamin E, chlorophylls, and quercetin) and nutritional substances (soluble proteins and soluble sugars), higher antioxidant activities (DPPH and FRAP), and higher levels of T-GLs, A-GLs, I-GLs, two aliphatic GLs (SIN and GNA), and two indolic GLs (GBC and 4ME) (Figure 5b). Interestingly, same cluster pairs were observed in pak-choi, while the indicators used to distinguish clusters were partially different. The cluster pair of CK, T400, and T380 was characterized by higher levels of I-GLs, A-GLs, T-GLs, two aliphatic GLs (GBN and RAA), three indolic GLs (4OH, NEO, GBC) and four minerals (Fe, Ca, Mg, and P) (Figure 5b). The cluster pair of T430 and T400 was similarly distinguished by better agronomy traits and enhanced phytochemicals like Chinese kale responses (Figure 5b).

Above results indicated a similar but also minor differential response pattern between CK and UV-A wavebands and between two blue wavebands in Chinese kale and pak-choi baby-leaves.

### 2.6. Multivariate Principal Component Analysis

**Figure 6:**
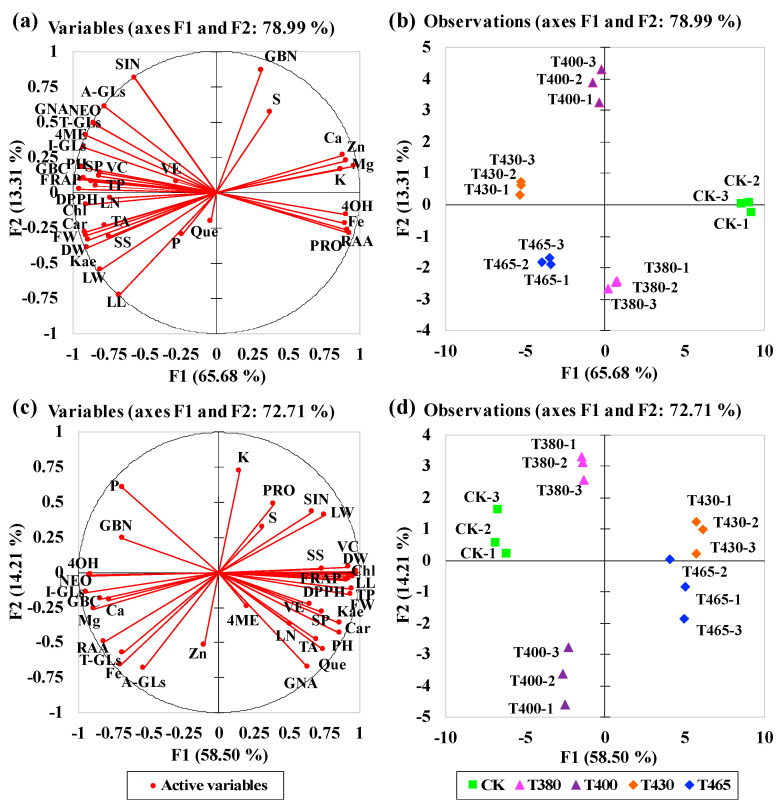
Multivariate principal component analysis showing the effects of supplementary blue and UV-A wavebands on two baby-leaves. The correlation circle of (**a**) Chinese kale and (**c**) Pak-choi. The scatter plot of (**b**) Chinese kale and (**d**) Pak-choi. FW = fresh weight, DW = dry weight, PH = plant height, LL = leaf length, LW = leaf width, LN = leaf number, Chl = chlorophylls, Car = carotenoids, TA = anthocyanins, VC = vitamin C, VE = vitamin E, TP = phenolics, Kae = kaempferol, Que = quercetin, DPPH = 2,2-diphenyl-1-picrylhydrazyl radical scavenging, FRAP = ferric reducing antioxidant power, SP = soluble proteins, SS = soluble sugars, A-GLs = aliphatic flucosinolates, I-GLs = indolic glucosinolates, T-GLs = total glucosinolates, PRO = progoitrin, RAA = glucoraphanin, SIN = sinigrin, GNA = gluconapin, GBN= glucobrassicanapin, 4OH = 4-hydroxyglucobrassicin, GBC = glucobrassicin, 4ME = 4-methoxyglucobrassicin, NEO = neoglucobrassicin, P = phosphorous, K = potassium, Ca = calcium, Mg = magnesium, S = sulphur, Zn = zinc, Fe = iron.

The principal component analysis (PCA) provided a global comparison of the correlations among all parameters tested in baby-leaves (Figure 6). The F1 explained 65.679% (Chinese kale) and 58.499% (pak-choi) of the total variability, whereas F2 explained 13.31% (Chinese kale) and 14.21% (pak-choi) of those. Therefore, F1 described the disparity among different light wavebands. The correlation circles showed the results were uneven in two baby-leaves (Figure 6). The longer vector length of the parameters could better represent F1 or F2. Meanwhile, the narrow angle between parameters depicted significant positive correlation, obtuse angle reflected notably negative correlation, and right angle described no relationship. For instance, in Chinese kale, very strong positive correlations were observed among vitamin C (VC), phenolics (TP), FRAP, DPPH, soluble proteins, plant height (PH), and GBC, and among carotenoids (Car), kaempferol (Kae), anthocyanins (TA), soluble sugars (SS), plant fresh weight (FW), and plant dry weight (DW) (Figure 6 a). Those indices were linked to axis F1 and had significant negative correlation with several GLs (RAA, PRO, and 4OH) and minerals (Fe, K, Mg, Zn, and Ca) (Figure 6a). In pak-choi, the 4OH, NEO, Ca, and Mg were found strong positive-related, which were negative linked to VC, DW, chlorophylls (Chl), leaf length (LL), TP, FW, and Kae (Figure 6c). Besides, in the PCA scatter plot, the response of two baby-leaves to T430 was not significantly different from T465: the most distinct reaction was to CK (Figure 6b,d).

## 3. Discussion

### 3.1. Improved Agronomy Traits of Baby-Leaves in Response to Different Blue and UV-A Wavebands

Light functions as the energy source and a primary signal regulating the plant growth, development, and metabolism. Different light wavelength could result in pronounced but variable effects on plants which strongly linked to species. In this study, blue (430 nm and 465 nm) and UV-A (380 nm and 400 nm) wavebands could significantly increase the biomass of two *Brassicaceae* baby-leaves, among which T430 and T465 were superior (Figure 1a,b). Similar effects were observed in blue light treated cabbage microgreens [34] and UV-A light treated cucumber plants [35]. However, neither kale, arugula, and mustard microgreens showed increased biomass under blue light [34], nor cucumbers under UV-B light [35]. One reason why blue wavebands were efficient in increasing the fresh and dry yield might be the enhanced PAR that help elevating stomatal opening and CO_2_ absorbing, enhancing the photosynthesis, and consequently increasing the plant biomass [35,36,37,38]. Besides, the photosynthetic pigments levels were closely linked to biomass accumulation. In this study, the content of chlorophylls was significant higher in both two baby-leaf plants supplemented with blue and UV-A wavebands, especially under T430 and T460 (Figure 2a,b), which was in consistent with the baby-leaves biomass accumulation.

Generally, blue light was considered as a negative regulate factor that inhibited gibberellin and auxin synthesis and/or sensitivity in plants and consequently suppressed the hypocotyl elongation [34,39,40,41], inter-node growth [42,43], and shoot length [44,45]. UV-A was also reported to a robust and dwarfed phenotype [35,44,46]. Differently in this study, Chinese kale and pak-choi baby-leaves exhibited higher plant height, enlarged leaf length, and remarkably increased leaf numbers under supplemental blue and UV-A light wavelengths (Figure 1c-f). These morphological changes might possibly contribute to the increased biomass, especially the increased leaf number which indicated an accelerated growth under blue and UV-A wavebands (Figure 1f). Similar effects induced by blue light were observed in the leaf length and petiole length of red-leaf and green-leaf pak-choi [47] and in the leaf number of green-leaved basil, purple-leaved basil, lamb’s lettuce, and garden rocket [48]. Notwithstanding the blue and UV-A wavebands could lead to similar changes in the agronomy traits of Chinese kale and pak-choi, the difference of the underlying mechanism still needs further study.

### 3.2. Elevated Pthytochemicals Contents of Baby-Leaves in Response to Different Blue and UV-A Wavebands

Vegetables are the main source of antioxidant compounds for human body such as vitamins, chlorophylls, vitamins, β-carotene, flavonoids, and polyphenols [49,50,51,52]. Human body inability to synthesize chlorophylls and carotenoids required a regular ingestion of them from vegetables [53]. In this study, both blue (T430 and T465) and UV-A lights (T380 and T400) markedly induced the chlorophylls and carotenoids accumulation in Chinese kale and pak-choi baby-leaves (Figure 2a,b). Among all, T430 was superior in increasing chlorophylls, while T465 led to the peak of carotenoids (Figure 2a,b). The PCA analysis showed that chlorophylls and carotenoids positively and strongly contributed to the baby-leaves antioxidant activity (DPPH and FRAP) (Figure 6a,b). Thus, the increased chlorophylls and carotenoids in two baby-leaves indicated a similar response to blue and UV-A lights. Studies in in broccoli sprouts also found that UV-A and UV-B were efficient in elevating the contents of individual carotenoid and chlorophyll [30]. And studies in basil, lettuce, and rocket leafy greens suggested that blue light could increase the chlorophyll synthesis [48].

Vitamins does not provide energy, and were not the component of cells, but a lack of vitamins can cause diseases such as scurvy, beriberi and rickets [54,55,56]. Similar to chlorophylls and carotenoids, vitamins cannot be synthesized in human body. In this study, the accumulation of VC was more pronouncedly triggered by blue lights (T430 and T465) than UV-A lights (T380 and T400) in two baby-leaves (Figure 2c). However, only T430 remarkably increased the VE content in pak-choi (Figure 2d). Regarding previous studies, blue light unaffected the VC contents in red-leaf and green-leaf lettuce [57], but increased the content of total L-ascorbic acid in leaf lettuce, spinach, and komatsuna [58]. And the UV-A light was found to improve the content of ascorbic acid in lettuce [59]. These results indicated the effects of light wavebands on vitamins were dependent on species.

Phenolics, flavonoids, and anthocyanins were important secondary metabolites that derived from phenylpropanoids, playing crucial role in plant defense system [60,61]. In one branch of the flavonoid biosynthesis pathway, the p-Coumaroyl-CoA can be catalyzed to kaempferol by a series of key enzymes, then the kaempferol can be catalyzed to quercetin [62,63]. Previous studies observed that the light could induce the phenolics, flavonoids (mainly quercetins) accumulation in faba bean [64] and lettuce [28], and anthocyanins accumulation in grape [65,66], kale [67], asparaguses [68], and radish [69]. Moreover, UV-B, UV-A, and blue light were found to trigger the gene expressions of the key enzymes related to the formation of these metabolites [70,71,72], through similar mechanisms mediated by photoreceptors such as UVR8, phototropins, and cryptochromes [28,64,65,71,72], as well as the transcript factors like *PIF*, *HY5*, and *MYB* families [73]. In line with these results, we found that the contents of phenolics and kaempferol were pronouncedly elevated by both blue and UV-A light, especially T430 (Figure 2f,g). Inversely, the quercetin content in Chinese kale sharply decreased under T380 and T400. Furthermore, the heatmap and PCA plot indicated strong positive correlation between antioxidant compounds and antioxidant activity, which could better explain the value of blue and UV-A wavebands in elevating the accumulation of functional phytochemicals of Chinese kale and pak-choi.

### 3.3. Altered Glucosinolates Levels of Baby-Leaves in response to Different Blue and UV-A Wavebands

**Figure 7:**
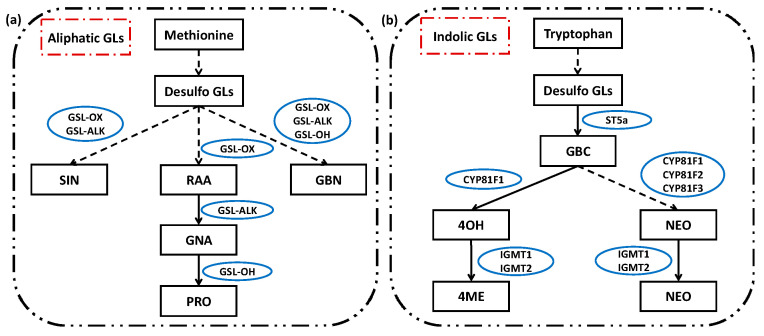
The simplified framework of aliphatic and indolic glucosinolate biosynthesis (modified based on Augustine et al. [79]). (**a**) Methionine-derived aliphatic glucosinolate and (**b**) Tryptophan-derived indolic glucosinolate. PRO = progoitrin, RAA = glucoraphanin, SIN = sinigrin, GNA = gluconapin, GBN= glucobrassicanapin, 4OH = 4-hydroxyglucobrassicin, GBC = glucobrassicin, 4ME = 4-methoxyglucobrassicin, NEO = neoglucobrassicin.

The GLs are a class of amino acid-derived, sulphur-rich secondary metabolites found in *Brassicaceae* vegetables [74], with a typical core structure containing a β-D-thioglucoside, a sulphonated aldoxime moiety, and a variable side chain derived from amino acids [75]. In the present study, five aliphatic GLs and four indolic GLs were identified and quantified in both two baby-leaves (Figure 4). The allyl-isothiocyanates (ITCs) and sulforaphane are two groups of the potent antioxidants and were hydrolyzed from SIN and RAA, respectively [17]. As shown in Figure 7a, RAA can be catalyzed into GNA which contributed to the spicy flavor of *Brassicaceae* vegetables [76,77]. Then, GNA was transformed into PRO which associated with the bitter flavor [77]. However, higher ingestion of PRO might lead to thyromegaly [77,78]. Besides, the indole-3-carbinol (I3C) is another promising chemopreventive compound that hydrolyzed from GBC [17]. Thus, SIN, RAA, and GBC were considered as the beneficial GLs, while PRO was regarded as the harmful GLs.

In the present study, blue and UV-A lights reduced the accumulation of PRO in Chinese kale, and the most effective wavebands were T400 (55.68%), T430 (55.14%), and T465 (52.75%), followed by T380 (30.65%) (Figure 4d). However, in pak-choi the PRO was significantly increased by T380 (20.15%), followed by T430 (10.79) and T465 (10.42%) (Figure 4e). These results reflected a differential PRO biosynthesis or degradation between two species in response to blue and UV-A lights. Concerning species, Chinese kale had higher profiles of three beneficial GLs than pak-choi (Figure 4d,e). Chinese kale also seemed to be more sensitive to blue and UV-A lights because of the obviously increased A-GLs, I-GLs, and T-GLs (Figure 4a–c).

In addition, the GBC and RAA were the predominant beneficial GLs in Chinese kale and pak-choi, respectively. The RAA accumulation was statistically retarded by different light wavebands in Chinese kale (60.67–87.62%) and in pak-choi (14.57–37.00%), while only T400 promoted the RAA content in pak-choi (Figure 4d,e). The GBC accumulation in Chinese kale was highly improved by T430 (694.87%), followed by T465 (493.46%), T400 (332.65%), then T380 (211.95%). Differently, the GBC content in pak-choi was markedly decreased by blue light (26.13–36.02%) but significantly increased by T380 (18.37%). Besides, the GNA content of two baby-leaves greatly increased, suggesting a stronger spicy flavor. Furthermore, the level of SIN, another beneficial GLs, in Chinese kale was pronouncedly heightened by T400 (205.34%), followed by T430 (150.27%), then T465 (68.09%). In contrast, T400 had no influence on the SIN of pak-choi, whereas T380 (9.33%) and T430 (10.67%) performed better in increasing the SIN content, followed by T465 (6.93%). From the whole, when elevating the beneficial GLs, T400 and T430 could be the best choice in Chinese kale, so as T400 in pak-choi.

### 3.4. Altered Minerals Contents of Baby-Leaves in Response to Different Blue and UV-A Wavebands

Minerals are indispensable components of cellular structures and co-enzymes and play a vital role in plant metabolism [80,81,82]. P and K are excellent sources of plant nutrition and benefit physiological processes [83]. Ca tends to accumulate in more mature parts of the plant while Mg are related to the initial developmental stages of vegetables [84]. In the present study, the contents of P, K, and S in pak-choi were not affected by different wavebands, while other minerals were found both increase and decrease in two baby-leaves under different wavebands (Figure 5). With the help of PCA and heatmap analysis, the similar response pattern of minerals and other indices in response to blue and UV-A light were easier to obtain (Figure 6 and Figure 7). Such as, lower content of Fe was characterized by T430 and T465 in Chinese kale, and the strong positive correlation between Fe and PRO/RAA/4OH and strong negative relationship between Fe and GBC/FRAP/DPPH/plant height (PH)/soluble proteins (SP) were observed. Although the accumulation pattern could be classified, the underlying mechanism still needs systematic studies.

## 4. Materials and Methods

### 4.1. Plant Material and Growth Conditions

**Figure 8:**
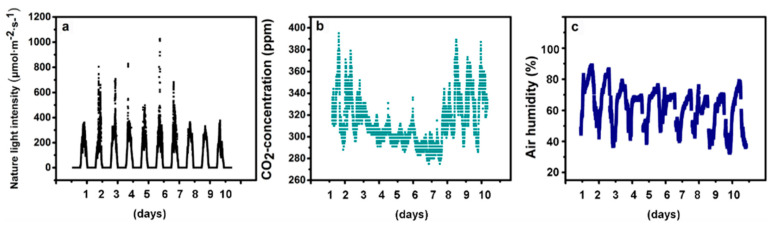
Environmental conditions of the greenhouse. The details of (**a**) the nature light intensity, (**b**) the CO_2_ concentration, and (**c**) the air humidity of the greenhouse.

This study was performed in a greenhouse of College of Horticulture, South China Agricultural University (longitude 113.36°E; latitude 23.16°N), with Chinese kale (*Brassica alboglabra* Bailey) and pak-choi (*Brassica campestris* L. ssp. *chinensis* Makino). After one hour of soaking, the seeds were sown in a matrix of coco coir and peat (1:1, *v:v*) and kept in dark at 25 ± 2 °C until germination. Then, the baby-leaves were cultivated with nutrient solution (CaNO_3_ 236.25 mg·L^−1^, KNO_3_ 151.75 mg·L^−1^, NH_4_PO_4_ 28.75 mg·L^−1^, MgSO_4_ 123.25 mg·L^−1^, pH = 6.0). The environment conditions in the green house were as follow: photosynthetic photon flux density (PPFD) at 12:00 AM, 400–1000 μmol·m^−2^·s^−1^ (Figure 8a); CO_2_ concentration, 270–400 ppm (Figure 8b); air humidity, 35–90% (Figure 8c), and 35℃/20℃ (day/night). After ten days, the blue and UV-A LED lights were supplemented using the wavelengths of 465 nm (T465), 430 nm (T430), 400 nm (T400), and 380 nm (T380), 12 h daily (6:00–18:00) at 40 W·m^−2^. And the control (CK) plants grown under the green house light without additional artificial light.

### 4.2. Agronomy traits Measurements

**Figure 9:**
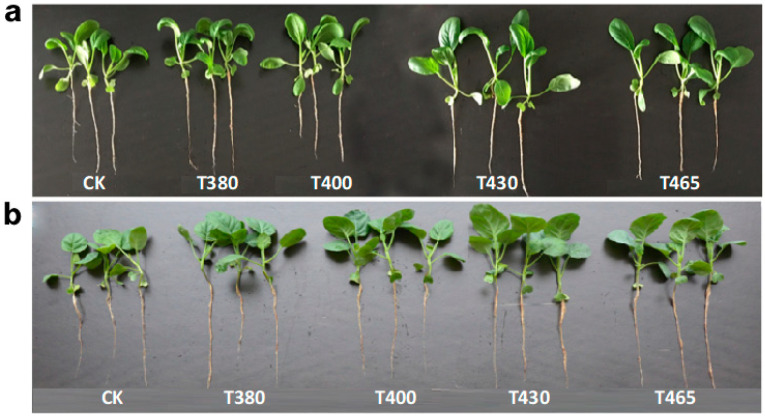
The two baby-leaves morphology 10 days after treatments. The morphology of (**a**) Pak-choi and (**b**) Chinese kale. CK = control. T380, T400, T430, and T465 = supplemented wavelength at 380, 400, 430, and 465 nm.

After ten days of treatment, the baby-leaf plants were sampled for measurements (Figure 9). The leaf number was recorded. The plant height, leaf length, and leaf width of the Chinese kale and pak-choi baby leaves were measured using a straightedge. The shoot of two baby-leaves were sampled for the fresh weight (FW) and dry weight (DW) measurements. The DW was determined after 48 h at 70 °C in a drying oven. Three biological repetitions were used in this study. Each biological repetition contained nine plants.

### 4.3. Phytochemicals Measurements

The contents of chlorophylls (Chl) and carotenoids (Car) measured colorimetrically [12]. Five hundred milligrams of fresh leaf tissue were extracted with 95% ethanol (*v:v*) and measured at 645 nm, 663 nm and 440 nm by using a UV spectrophotometer (Shimadzu UV-16A, Shimadzu, Corporation, Kyoto, Japan). The contents were calculated as follow:

Chlorophylls (mg·g^−1^) = (8.02 × OD_663_ + 20.20 × OD_645_) × V/(1000 W), (1)

Carotenoids (mg·g^−1^) = (4.70 × OD_440_ − 2.17 × OD_663_ − 5.45 × OD_645_) × V/(1000 W). (2)

The anthocyanin (TA) content was measured by the pH-differential spectrophotometry method [85]. Five grams of fresh sample was extracted with 25 mL of pH 1.0 buffer (50 mM KCl and 150 mM HCl) as well as 25 mL of pH 4.5 buffer (50 mM sodium acetate and 240 mM HCl). Absorbance was measured at 510 nm and 700 nm by a spectrophotometer, using a molar extinction coefficient of 24.825 for cyanidin-3-glucoside. The contents were calculated as follow:

Anthocyanins (mg·g^−1^) = [(OD_510_ − OD_700_) × 484.8]/24.825. (3)

The vitamin C (VC) content was determined according to the method reported by Kampfenkel et al. [86]. One gram of fresh shoot was ground and extracted with 5 mL of 5% trichloroacetic acid (TCA, *v:v*) and then centrifuged at 10,000 g for 10 min at 4 °C. The crude extract (1 mL) was added to 1 mL 5% TCA, 1 mL of 100% ethanol, 0.5 mL 0.4% H_3_PO_4_ (*w:v*), 1 mL of 0.5% 1,10-phenanthroline hydrate (*w:v*), and 0.5 mL 0.03% FeCl_3_ (*w:v*). Then the mixture was incubated at 30 °C for 1 h. The absorbance was recorded at 534 nm by using a UV spectrophotometer.

The vitamin E (VE) content was measured according to Prieto et al. [87]. The freeze-dried samples (0.2 g) were extracted with 8 mL ethanol and then 80 °C water bath for 1 h. After cooling to room temperature, the extract was centrifuged at 3000 g for 15 min. The supernatant (1 mL) was mixed with 2.5 mL ethanol. Then, 0.5 mL 6.0013 mM bathophenanthroline solution and 0.5 mL 1.0011 mM FeCl_3_ were added in order. After 15 s, 0.5 mL 4.0125 mM H_3_PO_4_ was added to quit reaction. Three minutes later, the absorbance was measured at 510 nm using a UV spectrophotometer.

The phenolics (TP) was determined colorimetrically [88]. Folin-Ciocalteu reagent was used with gallic acid as a positive control. Fresh samples (3.0 g) were homogenized with 25 mL ethanol for 30 min in darkness. After 15 min of being centrifuged at 3000 rpm, the supernatant (0.5 mL) was mixed with 2.5 mL of a of Folin-Ciocalteu reagent. After adding 2 mL 7.5% Na_2_CO_3_ (*w:v*), the mixture was kept at 45 °C for 15 min. The absorbance was measured at 765 nm by using a UV spectrophotometer.

The kaempferol (Kae) and quercetin (Que) were determined according to Miean and Mohamed [89]. The freeze-dried samples (0.2 g) were extracted with 2 mL 80% methanol (*v:v*) and 4 mL 2 M HCl. After 70 °C water bath for 40 min, 100% methanol (4 mL) was added then centrifuged at 10,000 g for 20 min at 4 °C. The supernatant filtered through a 0.22 μm organic phase filter membrane into the vials. The HPLC analysis used Agilent Technologies 1100 Series HPLC system equipped with a quaternary pump, an in-line degasser, a temperature controlled (5 °C) autosampler, a column heater and a photodiode array detector (DAD). The HPLC column was Thermo Hypersil-Keystone C18 Betasil, 250 mm × 2.1 mm i.d., 5 µm particle size (Thermo Fisher Scientific, Inc., Waltham, MA, USA). The HPLC conditions were as follow: the mobile phase A 100% methanol; the mobile phase B ultrapure water; the flow rate at 1.0 mL/min; the injection volume of 10 µL; UV chromatograms recorded at 368 nm.

The 2,2-diphenyl-1-picrylhydrazyl (DPPH) radical scavenging assay was carried out according to the method reported by Tadolini et al. [88]. Fresh tissue (3.0 g) was mixed with 25 mL methanol at 4 °C for 12 h and then centrifuged at 10,000 g for 10 min. The supernatants were used to prepare three types of mixture (Ai: Supernatant of 2 mL mixed with 2 mL 0.2 μM DPPH; Aj: Supernatant of 2 mL mixed with 2 mL ethanol; Ac: 0.2 μM DPPH mixed with 2 mL ethanol). The absorbance was read at 517 nm. The inhibition of DPPH free radical percentage was calculated as follows:

DPPH (%) = [1 − (Ai − Aj)/Ac] × 100%.(4)

The ferric-reducing antioxidant power (FRAP) assay was based on the method proposed by Thaipong et al. [90]. Three grams of fresh tissue were mixed with 25 mL methanol at 4 °C for 12 h and then centrifuged at 10,000 g for 10 min. The supernatants (0.4 mL) were mixed with 5.6 mL FRAP reagent (1.8 mL) and incubated in the 37 °C water bath for 10 min. The absorbance was measured at 593 nm by UV spectrophotometry.

The soluble proteins (SP) were determined according to Blakesley and Boezi [91]. Fresh samples (1.0 g) were extracted with 8 mL ultrapure water and centrifuged at 8000 g for 10 min. The supernatant (1 mL) was mixed with 5 mL 0.1 g·L^−1^ Coomassie brilliant blue G-250 solution. The absorbance was measured at 595 nm by a UV-spectrophotometer.

The soluble sugars (SS) measurement were performed according to Kohyama and Nishinari [91]. Fresh samples (1.0 g) were extracted with 10 mL 80% ethanol (*v:v*) and then added with 10 mg activated carbon powder. After 80 °C water bath for 40 min, the extract was diluted to a total volume of 25 mL with 80% ethanol (*v:v*). Then 0.2 mL mixture was mixed with 0.8 mL ultrapure water and 5 mL sulfuric acid anthrone reagent. After 100 °C water bath for 10 min, the absorbance at 625 nm was detected by the UV-spectrophotometer.

### 4.4. Glucosinolates Measurements

The glucosinolates (GLs) were determined using HPLC according to Grosser and Van Dam [92]. Freeze-dried samples (0.2 g) were extracted with 4 mL 70% MeOH and kept in boiling water bath and ultrasonic bath for 15 min. After ultra-sonication, the extracts were centrifuged at 5000 g and 4 °C for 10 min. The supernatants (5 mL) were mixed with 500 μL 500 mg·L^−1^ sulfatase solution, and then kept in the Pasteur pipette columns (filled with 500 μL DEAE-Sephadex A-25). After 12 h (in dark), the desulfoglucosinolates solution was eluted with 2 mL ultrapure water for HPLC. The sinigrin was used as the internal standard. The HPLC analysis was performed using the Merck-Hitachi HPLC system (Merck-Hitachi Ltd., Tokyo, Japan) consisting of a variable UV detector set at 227 nm and a Lichosphere RP-18 column (25 cm × 0.4 cm, 5 mm particle size; Merck, Darmstadt, Germany). The HPLC conditions were as follow: the mobile phase A ultrapure water; the mobile phase B 100% acetonitrile; gradient program, 0–32 min 0–20% B, 33–38 min 20% B, 39–40 min 20–100% B; the flow rate at 1.0 mL/min; the injection volume of 20 µL; temperature, 30 °C; UV chromatograms recorded at 229 nm.

### 4.5. Minerals Measurements

The minerals measurement was performed according to Waterland [80]. Oven-dried samples (0.5 g) were crushed into powder and ashed at 55 °C for 16 h. Then, 4 mL 70% HNO_3_ (*v:v*) was added for reaction. Four hours later, the extract was filtered and adjusted to a total volume of 20 mL with deionized distilled water. Mineral concentrations were determined by inductively coupled plasma spectrometry (Optima 2100DV; Perkin Elmer Corp., Waltham, MA, USA).

### 4.6. Statistical Analysis

Significant differences (*p* < 0.05) were determined by SPSS 25.0 software, using one-way analysis of variance (ANOVA) with Duncan’s multiple-range test. The multivariate principal component analysis (PCA) was performed with XLSTAT 2019 software (Addinsoft, v2019.2.2, New York, NY, USA, 2019). The cluster heatmap was generated by TBtools software [93].

## 5. Conclusions

In general, significant altered agronomic traits, phytochemical profiles, and mineral contents were observed in two *Brassicaceae* baby-leaves (Chinese kale and pak-choi) in response to supplementary blue and UV-A wavebands (380 nm, 400 nm, 430 nm, and 465 nm). Although four light wavebands could significantly accelerate the growth process of both baby-leaf plants, resulting in significant higher biomass, better morphological traits, and stronger antioxidant capacities, the blue wavebands at 430 nm and 465 nm were much more superior. Specifically, for the purpose of enhancing the contents of beneficial glucosinolates, 400 nm and 430 nm were highly recommended in Chinese kale and 400 nm was more suitable for pak-choi.
